# Migration and first-year maternal mortality among HIV-positive postpartum women: A population-based longitudinal study in rural South Africa

**DOI:** 10.1371/journal.pmed.1003085

**Published:** 2020-03-31

**Authors:** Hae-Young Kim, Adrian Dobra, Frank Tanser

**Affiliations:** 1 Africa Health Research Institute, KwaZulu-Natal, South Africa; 2 KwaZulu-Natal Research Innovation and Sequencing Platform (KRISP), KwaZulu-Natal, South Africa; 3 Department of Population Health, New York University School of Medicine, New York, New York, United States of America; 4 Department of Statistics, University of Washington, Seattle, Washington, United States of America; 5 Lincoln Institute for Health, University of Lincoln, Lincoln, United Kingdom; 6 School of Nursing and Public Health, University of KwaZulu-Natal, Durban, South Africa; 7 Centre for the AIDS Programme of Research in South Africa (CAPRISA), University of KwaZulu-Natal, KwaZulu-Natal, South Africa; Columbia University Mailman School of Public Health, UNITED STATES

## Abstract

**Background:**

In South Africa, within-country migration is common. Mobility affects many of the factors in the pathway for entry to or retention in care among people living with HIV. We characterized the patterns of migration (i.e., change in residency) among peripartum women from rural South Africa and their association with first-year postpartum mortality.

**Methods and findings:**

All pregnant women aged ≥15 years were followed-up during pregnancy and the first year postpartum in a population-based longitudinal demographic and HIV surveillance program in KwaZulu-Natal, South Africa, from 2000 to 2016. During the household surveys (every 4–6 months), each household head was interviewed to record demographic components of the household, including composition, migration, and mortality. External migration was defined as moving (i.e., change in residency) into or out of the study area. For women of reproductive age, detailed information on new pregnancy and birth was recorded. Maternal death was ascertained via verbal autopsy and HIV status at delivery via annual HIV surveys. We fitted mixed-effects Cox regression models adjusting for multiple pregnancies per individual. Overall, 19,334 women had 30,291 pregnancies: 3,339 were HIV-positive, 10,958 were HIV-negative, and 15,994 had unknown HIV status at delivery. The median age was 24 (interquartile range: 20–30) years. During pregnancy and the first year postpartum, 64% (*n* = 19,344) and 13% (*n* = 3,994) did not migrate and resided within and outside the surveillance area, respectively. Of the 23% who had externally migrated at least once, 39% delivered outside the surveillance area. Overall, the mortality rate was 5.8 per 1,000 person-years (or 831 deaths per 100,000 live births) in the first year postpartum. The major causes of deaths were AIDS- or tuberculosis-related conditions both within 42 days of delivery (53%) and during the first year postpartum (62%). In this study, we observed that HIV-positive peripartum women who externally migrated and delivered outside the surveillance area had a hazard of mortality more than two times greater (hazard ratio = 2.74; 95% confidence interval 1.01–7.40, *p*-value = 0.047)—after adjusting for age, time period (before or after 2010), and sociodemographic status—compared to that of HIV-positive women who continuously resided within the surveillance area. Study limitations include lack of data on access to antiretroviral therapy (ART) care and social or clinical context at the destinations among mobile participants, which could lead to unmeasured confounding. Further information on how mobile postpartum women access and remain in care would be instructive.

**Conclusions:**

In this study, we found that a substantial portion of peripartum women moved within the country around the time of delivery and experienced a significantly higher risk of mortality. Despite the scale-up of universal ART and declining trends in maternal mortality, there is an urgent need to derive a greater understanding of the mechanisms underlying this finding and to develop targeted interventions for mobile HIV-positive peripartum women.

## Abstract 2

In a study done in South Africa, Hae-Young Kim and colleagues investigate migration and maternal mortality in women with HIV infection.

## Author summary

**Why was this study done?:**

- Within-country migration is common among young women, often seeking economic or educational opportunities at nearby cities.
- Migration is a risk factor for poor retention in care among people living with HIV. HIV-positive women who initiate or continue lifelong antiretroviral therapy (ART) during pregnancy face unique challenges to remain in HIV care in the postpartum period, potentially leading to worse health outcomes.
- Little is known about within-country mobility patterns among peripartum women and their link to maternal health outcomes.

**What did the researchers do and find?:**

- Using one of the largest population-based longitudinal HIV and demographic surveys (2000–2016), we characterized the patterns of external migration (i.e., change of residency into or away from the study area) among peripartum women from rural KwaZulu-Natal, South Africa, and examined the first-year risk of death by maternal HIV status and migration patterns.
- More than 20% had externally migrated at least once during pregnancy and in the first year postpartum; of these, about 40% delivered outside the study area. Overall, the maternal mortality rate was very high at 5.8 per 1,000 person-years (or 831 deaths per 100,000 live births) in the first year postpartum.
- HIV-positive peripartum women who externally migrated and delivered outside the study area had more than two times the hazard of mortality, compared to those who continuously resided within the surveillance area. However, such an association was not observed among HIV-negative peripartum women.

**What do these findings mean?:**

- In this study in rural South Africa, we found that a substantial portion of peripartum women migrates around the time of delivery. Mobile HIV-positive peripartum women experience poor health outcomes, including higher first-year mortality.
- Further understanding of how mobile HIV-positive postpartum women access and remain in care is critical to provide targeted interventions and improve maternal health outcomes.

## Introduction

South Africa has the largest number of people living with HIV (PLWH) worldwide, constituting 7.7 million in 2018, of whom 4.7 million (61%) were women aged 15 or older [1]. Women of reproductive age often receive their first HIV screening during antenatal care visits. In 2017, more than one in four pregnant women who visited public antenatal clinics were HIV-positive [2]. Since 2015, the South African government has adopted Option B+ as the official policy, which recommends initiation of lifelong antiretroviral therapy (ART) to pregnant women with HIV regardless of their CD4 counts [3]. This was successfully scaled up, with over 95% of HIV-positive pregnant women with HIV receiving ART at public antenatal care clinics in 2017 [2]. However, a few studies have shown that HIV-positive pregnant women who initiate ART during pregnancy are at increased risk of attrition and loss to follow-up in the postpartum period in South Africa [4–8] and other African countries [9].

One of the key factors affecting retention in care is migration. Frequent travel or circular movement has been associated with treatment interruption, nonadherence to ART, poor virologic suppression, and lower survival rates among PLWH [10–12]. In the 25 years since the advent of democracy in South Africa, within-country migration for temporary work and frequent visits to homes in rural areas have been common and uniquely contributed to the spread of HIV [13,14]. In a rural setting of KwaZulu-Natal, female migration, especially among young women aged 15–25 years, has increased over the years, and often destined to nearby cities [15,16]. Mobile populations face various challenges in accessing healthcare, such as identifying new clinics and arranging transports to visit clinics in a new environment, or lacking social support [17,18]. PLWH might also self-select into migration because of social stigma, further hindering their efforts to seek care [19].

HIV-positive peripartum women are particularly vulnerable to the potential consequences of migration. Several studies have reported that HIV-positive pregnant and postpartum women may return home to deliver or after delivery for family support and might experience disengagement in care [13,20,21]. Access to and retention in care are critical for both infant and maternal health as well as maternal adherence to lifelong ART. However, there have been limited in-depth studies on the mobility patterns among HIV-positive women around the time of delivery across the country and how mobility might be linked to their health outcomes.

The population-based longitudinal survey at the Africa Health Research Institute (AHRI) in the rural uMkhanyakude district in northern KwaZulu-Natal is one of the world’s largest population-based cohorts and follows up the movement of every individual registered in the surveillance site (*N* ≈ 90,000). In this study, we examined the mobility patterns of women during pregnancy and the first year postpartum and explored the association with postpartum mortality by maternal HIV status.

## Methods

### Study setting

The AHRI population-based longitudinal surveillance system contains comprehensive individual and household level data from 2000 to 2016, being located in an area with one of the highest HIV rates in South Africa. The adult HIV prevalence was estimated to be >30% in 2016 [22], and HIV prevalence among pregnant women in KwaZulu-Natal was 41.1% in 2017 [2].

A detailed description of this cohort has been published elsewhere [23]. Briefly, the surveillance covers over 90,000 household members from approximately 12,000 households in the 438-km^2^ demographic study area, with household response rates exceeding 99% [23]. Trained fieldworkers visit all households in the study area every 4–6 months. Individuals can freely move into or out of the study area. Previously, the main reasons for migrating out of the study area were seeking formal employment or education or changes in partnership [24,25]. Households are defined as social groups of individuals who mainly share the same resources, have one household head, and know the basic information about each other [23]. During household visits, each household head or key informant is interviewed regarding the demographic components of the household, including size, composition, residential status of members, fertility, and mortality, as well as sociodemographic information of household members such as education and marital status. Resident members are defined as those keeping their day-to-day belongings and sleeping at the homestead most nights, whereas nonresident members do not normally reside at the homestead but maintain social and physical connections via returned visits and support. Each individual is allowed to have only one place of residency at any given time. All resident members aged 15 years and older are eligible to receive annual HIV testing after completing written informed consent.

### Participants and procedures

We followed-up all pregnant women in the surveillance system with known residential status at the time of pregnancy up to 1 year after delivery from 2000 to 2016. When a woman was first enrolled in the surveillance, detailed information about her previous pregnancies was collected. At subsequent visits, information regarding any new pregnancy was recorded, including the last menstrual period, date of delivery, pregnancy outcome, and details of antenatal clinic attendance. The date of the last menstrual period was used as a proxy for the start date of pregnancy; if the date was missing, it was imputed as 40 weeks prior to the recorded delivery date.

### Exposures

The primary exposure was mobility patterns during pregnancy and the first year postpartum. We defined external migration as moving into or out of the surveillance area (i.e., changes in residential status from residency to nonresidency or vice versa). Those who did not externally migrate were stratified by having continuously resided within the surveillance area or outside the surveillance area. Participants who experienced external migration during the follow-up period were further stratified by delivering inside or outside of the surveillance area. Thus, we fitted a total of four different mobility patterns in the analysis.

### Outcome

The primary outcome was maternal mortality in the first year postpartum. The maternal mortality ratio is typically calculated from pregnancy-related maternal deaths during pregnancy or childbirth or within 42 days of termination of pregnancy, whereas later maternal deaths include deaths within 1 year after the end of pregnancy due to indirect and direct obstetric causes or management [26,27]. In this study, we included all maternal deaths that occurred in the first year postpartum, as it is the critical time for both infant and maternal health outcomes. Mortality was ascertained via verbal autopsy with the closest caregiver of the deceased on an average of 6 months after the person’s death [28]. During the interview, the caregiver was asked to provide a narrative of the circumstances related to the death of the individual. A checklist of signs and symptoms and a standard questionnaire adapted from the WHO/INDEPTH questionnaire for verbal autopsy were used [29,30]. Causes of deaths were determined by the InterVA-4 model according to the categories defined in the WHO 2012 Verbal Autopsy Instrument, which are compatible with the International Classification of Diseases, 10th revision (ICD-10) [31]. The InterVA-4 model is a probabilistic program that extracts the indicators from the verbal autopsy questionnaire and determines the most likely causes of deaths based on a set of symptoms, signs, and circumstances reported in verbal autopsy interviews [32–34]. It has been validated using the AHRI’s population-based data [28] as well as the surveillance data across five different African and Asian countries [35]. The InterVA model had 97% concordance with physician coding when HIV/AIDS and pulmonary deaths were combined together as the cause of deaths [35] and 90% specificity for identifying HIV/AIDS-related deaths among known perimortem HIV-positive individuals [36]. Thus, deaths due to tuberculosis (TB) or HIV/AIDS were classified together as HIV-related deaths, given the overlap of mortality and symptoms between the two diagnoses. More details on the verbal autopsy methods and validation have been published elsewhere [28].

### Covariates

Maternal HIV status at the time of delivery was ascertained via annual population-based HIV surveys, with the midpoint between the last HIV-negative test date and the first HIV-positive test date being used as a proxy for the date of HIV infection. History of antenatal care visits, delivery settings, sociodemographic information on the household asset, education, and marital status were included as potential risk factors. The AHRI surveillance is linked with the electronic records for ART initiation and management from patients attending the 17 clinics in the Hlabisa Health subdistrict via the probabilistic matching algorithms, with records matching prior to 2015 being suboptimal. Thus, we fitted a variable to indicate whether delivery occurred before or after 2010, the point at which the public ART program was scaled up at the population level. Pregnant or postpartum women often travel to their family members to receive social support and care [20,21]. Thus, we also included a variable to indicate whether either of the participant’s parents lived and were registered in the surveillance area. Age and year of delivery were fitted as continuous variables in the sensitivity analysis using spline terms (S1 Table).

### Sensitivity analysis

A substantial number of participants who were last known as HIV-negative, or had unknown HIV status during pregnancy, died in the postpartum period without any further HIV test. However, the causes of deaths for some individuals were recorded as attributable to AIDS or TB by the InterVA-4 model. To reduce potential bias due to missing data and misclassification, and to explore the rigorousness of the study results, we ran the sensitivity analysis regarding unknown HIV status, in which those whose deaths were attributable to HIV/AIDS or TB were assumed to have been HIV-positive prior to deaths, whereas the rest of mothers with unknown HIV status were assumed as HIV-negative (Method 2).

### Statistical analysis

**Fig 1:**
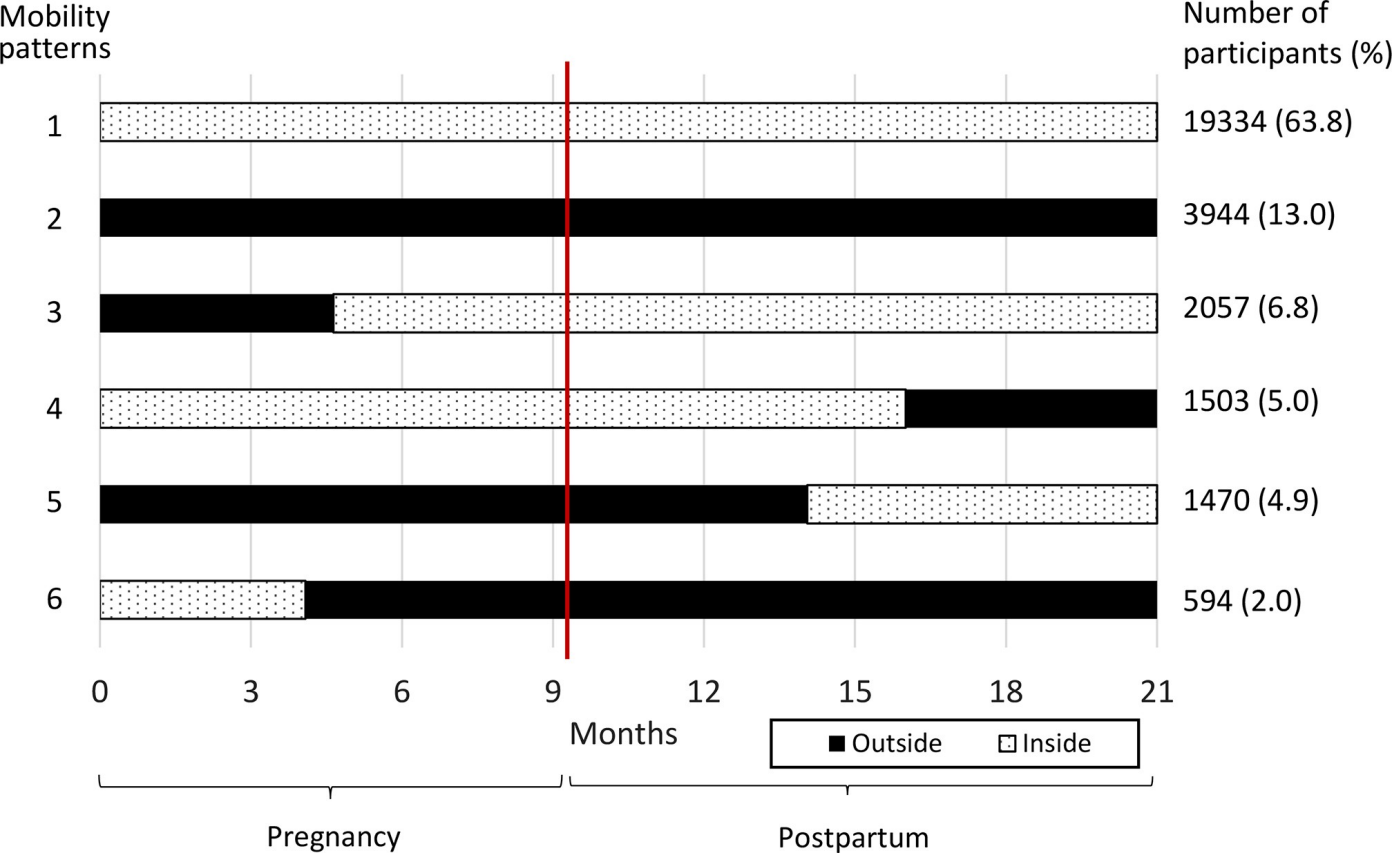
Mobility patterns of peripartum women during pregnancy and the first year postpartum. Black (“Outside”) and dotted (“Inside”) refer to the time period living outside and inside the surveillance area, respectively. The red line indicates the timing of delivery at 40 weeks of gestation. The bar length represents the average duration of residence outside or inside the demographic surveillance area in each mobility pattern. The figure shows the top six most frequent mobility patterns, accounting for >98% participants.

The average duration of residence within or outside the surveillance area during pregnancy and the first year postpartum is presented in Fig 1. The mortality rate was calculated as the number of maternal deaths per 1,000 person-years (PY) by maternal HIV status. We also report the number of maternal deaths per 100,000 live births within 42 days of delivery and in the first year postpartum to compare with the standardized mortality rates reported by WHO [27]. The mixed-effects Cox regression model was fitted to examine the association between mobility patterns and the first-year maternal mortality, accounting for repeated pregnancies per individual. All mothers were censored 365 days after delivery if they were alive or on the date of death if they died in the first year postpartum. The adjusted models included potential confounders such as age, parity, education, and household asset. We fitted interaction terms between maternal HIV status and mobility patterns in sensitivity analyses. For those who externally out-migrated, we geospatially mapped their destinations reported by the household members and calculated the distance between their destinations and the residential locations within the surveillance area prior to the migration. All analysis was conducted in STATA 15.0 and R 3.2.2. This study is reported as per the Strengthening the Reporting of Observational Studies in Epidemiology (STROBE) guidelines (see S1 STROBE Checklist) [37]. For further information regarding the prespecified analysis plan, see S1 Text.

### Ethics

Funding sources had no role in the decision to prepare the manuscript and submit it for publication. The permission for the demographic and HIV surveillance was approved by the University of KwaZulu-Natal Biomedical Research Ethics Committee (BE290/16).

## Results

### Baseline characteristics and mobility patterns

There was a total of 30,291 pregnancies among 19,334 women aged 15 years or older with known residential status at the time of pregnancy between 2000 and 2016. Almost all participants (98%) had one of the six mobility patterns as shown in Fig 1. Approximately two-thirds of pregnancies (64%, *n* = 19,334) occurred among the participants who did not migrate and resided in the surveillance area, whereas 13% (*n* = 3,994) resided outside the surveillance area as nonresidents during pregnancy and in the first year postpartum. Of the 23% (*n* = 3,944) who had at least one episode of external migration, 39% (*n* = 2,675) delivered outside the surveillance area. Table 1 shows the baseline characteristics at delivery by the four different mobility patterns. Overall, the median age was 24 (interquartile range [IQR]: 20–30) years, approximately 90% were never married, and 75% of the pregnant women’s parents were registered in the study area. Over 80% of the women delivered at hospitals or clinics. Maternal HIV status at delivery was ascertained for 47% of the pregnancies, with a substantially higher number of unknown HIV status among those who continuously resided outside the study area (S2 Table). When examined by maternal HIV status, HIV-positive pregnant women significantly had more parity and were older than the HIV-negative women or those with unknown HIV status (S3 Table).

**Table 1:** Baseline characteristics at delivery by the mobility patterns among 30,291 pregnant women registered in the population-based DSA, KwaZulu-Natal, South Africa.

	External Migration			
	No		Yes	
	Resided Within DSA	Resided Outside DSA	Delivery Within DSA	Delivery Outside DSA
	*n* = 19,334	*n* = 3,944	*n* = 4,338	*n* = 2,675
**Age at delivery,** Median (Q1, Q3) (years)	24 (20, 31)	26 (22, 30)	23 (20, 27)	24 (21, 28)
	*n* (%)	*n* (%)	*n* (%)	*n* (%)
**HIV status at delivery**				
Negative	8,526 (44.1)	533 (13.5)	1,253 (28.9)	646 (24.1)
Positive	2,479 (12.8)	238 (6.0)	397 (9.2)	225 (8.4)
Unknown	8,329 (43.1)	3,173 (80.5)	2,688 (62.0)	1,804 (67.4)
**Time period (years)**				
2000–2010	13,521 (69.9)	2,899 (73.5)	3,018 (69.6)	1,969 (73.6)
2011–2016	5,810 (30.1)	1,045 (26.5)	1,319 (30.4)	706 (26.4)
Ever attending ANC visits during the pregnancy*				
Yes	5,845 (30.2)	1,460 (37.0)	1,319 (30.4)	923 (34.5)
No	2,618 (13.5)	290 (7.4)	572 (13.2)	283 (10.6)
Unknown	10,871 (56.2)	2,194 (55.6)	2,447 (56.4)	1,469 (54.9)
**Delivery setting**				
Own home	1,298 (6.7)	99 (2.5)	233 (5.4)	121 (4.5)
Hospital	10,962 (56.7)	2,409 (61.1)	2,580 (59.5)	1,552 (58.0)
Clinics	4,794 (24.8)	643 (16.3)	1,031 (23.8)	512 (19.1)
Other or unknown	2,280 (11.8)	793 (20.1)	494 (11.4)	490 (18.3)
**Parity**				
0	7,457 (38.6)	1,524 (38.6)	2,038 (47.0)	1,205 (45.0)
1	4,565 (23.6)	1,206 (30.6)	1,217 (28.1)	763 (28.5)
2+	7,312 (37.8)	1,214 (30.8)	1,083 (25.0)	707 (26.4)
**Parents registered in the surveillance area**				
Yes	13,951 (72.2)	3,179 (80.6)	3,452 (79.6)	2,136 (79.9)
No	5,383 (27.8)	765 (19.4)	886 (20.4)	539 (20.2)
**Asset**				
Poorest	1,149 (6.0)	272 (6.9)	319 (7.4)	233 (8.8)
Poor	5,265 (27.3)	1,040 (26.5)	1,231 (28.5)	686 (25.8)
Medium	5,692 (29.5)	1,096 (27.9)	1,248 (28.9)	764 (28.7)
Rich or richest	7,165 (37.2)	1,515 (38.6)	1,519 (35.2)	977 (36.7)
**Education**				
No formal education or primary (grade <7)	3,590 (18.6)	479 (12.1)	649 (15.0)	425 (15.9)
Secondary (grade ≥8)	14,697 (76.0)	3,128 (79.3)	3,494 (80.5)	2,068 (77.3)
Missing	1,047 (5.4)	337 (8.5)	195 (4.5)	182 (6.8)
**Marital status**				
Married	2,200 (11.4)	214 (5.4)	132 (3.0)	97 (3.6)
Divorced, separated, widowed	197 (1.0)	19 (0.5)	26 (0.6)	10 (0.4)
Never married	16,557 (85.6)	3,636 (92.2)	4,150 (95.7)	2,542 (95.0)

Abbreviations: ANC, antenatal care; DSA, demographic surveillance area

### Maternal mortality rates and causes of deaths in the first year postpartum

Between 2000 and 2016, there were 40 deaths within 42 days of delivery and 215 in the first year postpartum, with the majority of the deaths (181/215, 84%) occurring before 2011. The mortality rate was 132 deaths per 100,000 live births within 42 days of delivery and 831 deaths per 100,000 live births in the first year postpartum. The overall mortality rate was 5.6 per 1,000 PY in the first year postpartum, with Table 2 showing the mortality rates by maternal HIV status and mobility patterns. The mortality rate among known HIV-negative women and HIV-positive women was 0.5/1,000 PY (95% CI 0.2–1.1) and 9.3/1,000 PY (95% CI 6.5–13.3), respectively. Of known HIV-positive postpartum women, those who continuously resided within or outside the surveillance area had mortality rates of 7.5/1,000 PY (95% CI 4.7–12.0) and 17.5/1,000 PY (95% CI 6.6–46.7), respectively. Of HIV-positive postpartum women who had externally migrated at least once, those who delivered within the surveillance or outside the surveillance area had mortality rates of 7.8/1,000 PY (95% CI 2.5–24.1) and 22.6/1,000 PY (95% CI 9.4–54.2), respectively. In the sensitivity analysis, when we assumed that the women with unknown HIV status whose deaths were attributable to AIDS or TB were HIV-positive, the overall mortality rate in HIV-positive women was 42.1/1,000PY (Table 2, Method 2).

**Table 2:** Mortality rate in the first year postpartum by maternal HIV status at delivery and mobility patterns.

	HIV-Negative			HIV-Positive			Unknown HIV Status		
	No. Deaths	PY	Rate per 1,000 PY* (95% CI)	No. Deaths	PY	Rate per 1,000 PY* (95% CI)	No. Deaths	PY	Rate per 1,000 PY* (95% CI)
Method 1*									
Resided within DSA	5	8,473	0.6 (0.2–1.4)	18	2,389	7.5 (4.7–12.0)	100	8,094	12.4 (10.2–15.0)
Resided outside DSA	0	531	0	4	228	17.5 (6.6–46.7)	46	3,073	15.0 (11.2–20.0)
Migration and delivery within DSA	0	1,247	0	3	386	7.8 (2.5–24.1)	20	2,613	7.7 (4.9–11.9)
Migration and delivery outside DSA	0	647	0	5	222	22.6 (9.4–54.2)	14	1,769	7.9 (4.7–13.4)
**Method 2**^**§**^									
Resided within DSA	46	16,536	2.7 (2.0–3.6)	77	2,418	31.8 (25.5–39.8)			
Resided outside DSA	23	3,595	6.4 (4.3–9.6)	27	237	113.9 (78.1–166.0)			
Migration and delivery within DSA	6	3,852	1.6 (0.7–3.5)	17	393	43.2 (26.9–69.5)			
Migration and delivery outside DSA	2	2,408	0.8 (0.2–3.3)	17	230	73.9 (46.0–118.9)			

*Women were stratified by their HIV status at the time of delivery.

§Women with unknown HIV status whose death were attributable to AIDS or TB were considered as HIV-positive and the rest of mothers with unknown HIV status as HIV-negative.

Abbreviations: DSA, demographic surveillance area; PY, person-years

The major cause of deaths was AIDS- or TB-related conditions both within 42 days of delivery (53%) and in the first year postpartum (62%), followed by maternal, perinatal, nutritional, or congenital causes (28%) within 42 days of delivery (Table 3).

**Table 3:** Causes of deaths in the first year postpartum after delivery*.

	Overall	Within 42 Days	Between 43 and 365 Days
Category	*N* (%)	*N* (%)	*N* (%)
AIDS or TB-related causes	129 (60)	21 (53)	108 (62)
Maternal, perinatal, nutritional, and congenital causes	30 (14)	11 (28)	19 (11)
Noncommunicable conditions	14 (7)	3 (8)	11 (6)
Injuries	8 (4)	1 (3)	7 (4)
Unknown	34 (16)	4 (10)	30 (17)

*The InterVA-4 model was used to determine the most likely causes of deaths.

Abbreviation: TB, tuberculosis

### Association between mobility patterns and maternal mortality in the first year postpartum

Table 4 shows the associations between mobility patterns and the first-year mortality among HIV-positive women. In the unadjusted mixed-effects Cox regression model, HIV-positive women who externally migrated and delivered outside the surveillance area had a hazard of mortality approximately three times that of women who resided within the surveillance area (hazard ratio [HR] = 3.01; 95% CI 1.11–8.11) (Table 4, Method 1). This association was still significant after adjusting for maternal age, time period, and socioeconomic status (adjusted HR [aHR] = 2.74, 95% CI 1.01–7.40). When the women with unknown HIV status whose deaths were attributable to AIDS- or TB-related causes were assumed to have been HIV-positive, the results were similar, although the association was slightly attenuated and was no longer statistically significant after adjusting for other covariates (aHR = 1.62; 95% CI 0.89–2.96) (Table 4, Method 2).

**Table 4:** Association between mobility patterns and mortality in the first year postpartum among HIV-positive women in 2000–2016.

	Method 1		Method 2^†^	
Characteristic	Hazard Ratio (95% CI)	Adjusted Hazard Ratio (95% CI)^‡^	Hazard Ratio (95% CI)	Adjusted Hazard Ratio (95% CI)^‡^
**Migration**				
Resided within DSA	Ref	Ref	Ref	Ref
Resided outside DSA	2.33 (0.79–6.89)	2.34 (0.79–6.95)	**3.72 (2.34**–5.93)**	**3.76 (2.29**–6.19)**
Migration and delivery within DSA	1.03 (0.30–3.51)	1.08 (0.32–3.67)	1.38 (0.81–2.37)	1.35 (0.76–2.40)
Migration and delivery outside DSA	**3.01 (1.11**–8.11)*	**2.74 (1.01**–7.40)*	**2.34 (1.35**–4.04)**	1.62 (0.89–2.96)
**Age (years)**				
<20	Ref	Ref	Ref	Ref
20–30	1.35 (0.31–5.83)	1.35 (0.31–5.87)	0.87 (0.48–1.56)	0.98 (0.51–1.88)
>30	1.17 (0.26–5.33)	1.35 (0.29–6.23)	0.70 (0.37–1.30)	1.15 (0.53–2.47)
**Time period (calendar year)**				
2000–2010	Ref	Ref	Ref	Ref
2011–2016	**0.34 (0.15**–0.79)*	**0.36 (0.15**–0.84)*	**0.10 (0.05**–0.18)**	**0.14 (0.08**–0.27)**
**Delivery setting**				
Own home	Ref		Ref	Ref
Hospital	0.68 (0.20–2.29)		**0.54 (0.30**–0.96)*	0.87 (0.47–1.60)
Clinics	0.22 (0.05–1.11)		**0.36 (0.18**–0.70)**	0.51 (0.25–1.03)
Other or unknown	0.61 (0.12–3.01)		0.66 (0.32–1.38)	**0.43 (0.19**–0.96)*
**Parity**				
0	Ref		Ref	Ref
1	0.80 (0.30–2.15)		0.76 (0.48–1.20)	0.65 (0.39–1.10)
2+	0.70 (0.28–1.73)		**0.59 (0.38**–0.90)*	**0.52 (0.29**–0.94)*
**Education**				
Secondary+ (grade ≥8) versus grade <8	1.04 (0.40–2.72)		**0.57 (0.37**–0.88)*	0.91 (0.57–1.46)
**Socioeconomic status (household asset)**				
Poorest	Ref	Ref	Ref	Ref
Poor	0.51 (0.16–1.58)	0.51 (0.16–1.60)	**0.16 (0.11**–0.25)**	**0.25 (0.16**–0.39)**
Medium	0.41 (0.13–1.30)	0.43 (0.14–1.35)	**0.09 (0.05**–0.14)**	**0.14 (0.08**–0.23)**
Rich or richest	**0.11 (0.02**–0.49)**	**0.12 (0.03**–0.52)**	**0.03 (0.01**–0.06)**	**0.05 (0.02**–0.10)**

Statistically significant *p*-values (<0.05) are in bold font.

*p-value < 0.05.

**p-value < 0.01.

†Women with unknown HIV status whose death were attributable to AIDS or TB were considered as HIV-positive.

‡Adjusted for all other covariates shown in each column.

Abbreviations: DSA, demographic surveillance area; TB, tuberculosis

We further examined the effect of mobility patterns on mortality among all HIV-positive and HIV-negative pregnant women (Table 5). Among HIV-negative women, residing outside the surveillance area was associated with a higher hazard of mortality (aHR = 1.93, 95% CI 1.11–2.32, *p*-value = 0.01), whereas external migration and delivery outside the surveillance area were not associated with mortality (aHR = 0.79, 95% CI 0.45–1.40, *p*-value = 0.40). In contrast, external migration and delivery outside the surveillance area were associated, among HIV-positive women, with a hazard of mortality (aHR = 3.41; 95% CI 1.07–10.82) more than three times that of HIV-negative women (Table 5, Method 1). When we assumed the women with unknown HIV status whose deaths were attributable to AIDS or TB to be HIV-positive and the rest of mothers with unknown HIV status to have been HIV-negative at delivery (thereby including all eligible pregnant women in the analysis), this association became stronger (aHR = 7.77; 95% CI 1.71–35.19). HIV-negative postpartum women who externally migrated and delivered outside the surveillance area had a nonsignificantly lower risk of mortality, compared to HIV-negative women who continuously resided in the surveillance area (aHR = 0.25; 95% CI 0.06–1.01) (Table 5, Method 2).

**Table 5:** Association between mobility patterns and mortality in the first year postpartum among all eligible women in 2000–2016.

	Method 1^†^	Method 2^‡^
Characteristic	Adjusted Hazard Ratio (95% CI)^§^	Adjusted Hazard Ratio (95% CI)^§^
**HIV status**		
Negative	Ref	Ref
Positive	1.27 (0.76–2.12)	**12.85 (8.82**–18.73)**
**Migration**		
Resided within DSA	Ref	Ref
Resided outside DSA	1.74 (1.22–2.50)	**1.93 (1.16**–3.20)*
Migration and delivery within DSA	0.73 (0.45–1.20)	0.49 (0.21–1.16)
Migration and delivery outside DSA	0.79 (0.45–1.40)	0.25 (0.06–1.01)
**HIV-positive status × migration**^¶^		
Positive × reside outside DSA	1.36 (0.43–4.32)	1.81 (0.92–3.55)
Positive × migration and delivery within DSA	1.47 (0.39–5.54)	2.56 (0.93–7.02)
Positive × migration and delivery outside DSA	**3.41 (1.07**–10.82)*	**7.77 (1.71**–35.19)**
**Age (years)**		
<20	Ref	Ref
20–30	**2.53 (1.57**–4.08)**	**2.01 (1.21**–3.35)**
>30	**2.99 (1.80**–4.96)**	**2.81 (1.54**–5.11)**
**Time period (calendar year)**		
2000–2010	Ref	Ref
2011–2016	**0.47 (0.32**–0.70)**	**0.29 (0.20**–0.43)**
**Parity**		
0		Ref
1		0.71 (0.49–1.04)
2+		**0.53 (0.36**–0.80)**
**Socioeconomic status (household asset)**		
Poorest	Ref	Ref
Poor	**0.24 (0.17**–0.34)**	**0.23 (0.17**–0.32)**
Medium	**0.12 (0.08**–0.18)**	**0.11 (0.08**–0.17)**
Rich or richest	**0.07 (0.05**–0.11)**	**0.07 (0.05**–0.11)**

Statistically significant *p*-values (<0.05) are in bold font.

*p-Value < 0.05.

**p-Value < 0.01.

**†**Women with unknown HIV status were considered as HIV-negative.

‡Women with unknown HIV status whose death were attributable to AIDS or TB were considered as HIV-positive and the rest of mothers with unknown HIV status as HIV-negative.

§Adjusted for all other covariates shown in the column.

¶Interaction terms between HIV status and migration patterns.

Abbreviations: DSA, demographic surveillance area; TB, tuberculosis

### Mobility among those who out-migrated from the surveillance

**Fig 2:**
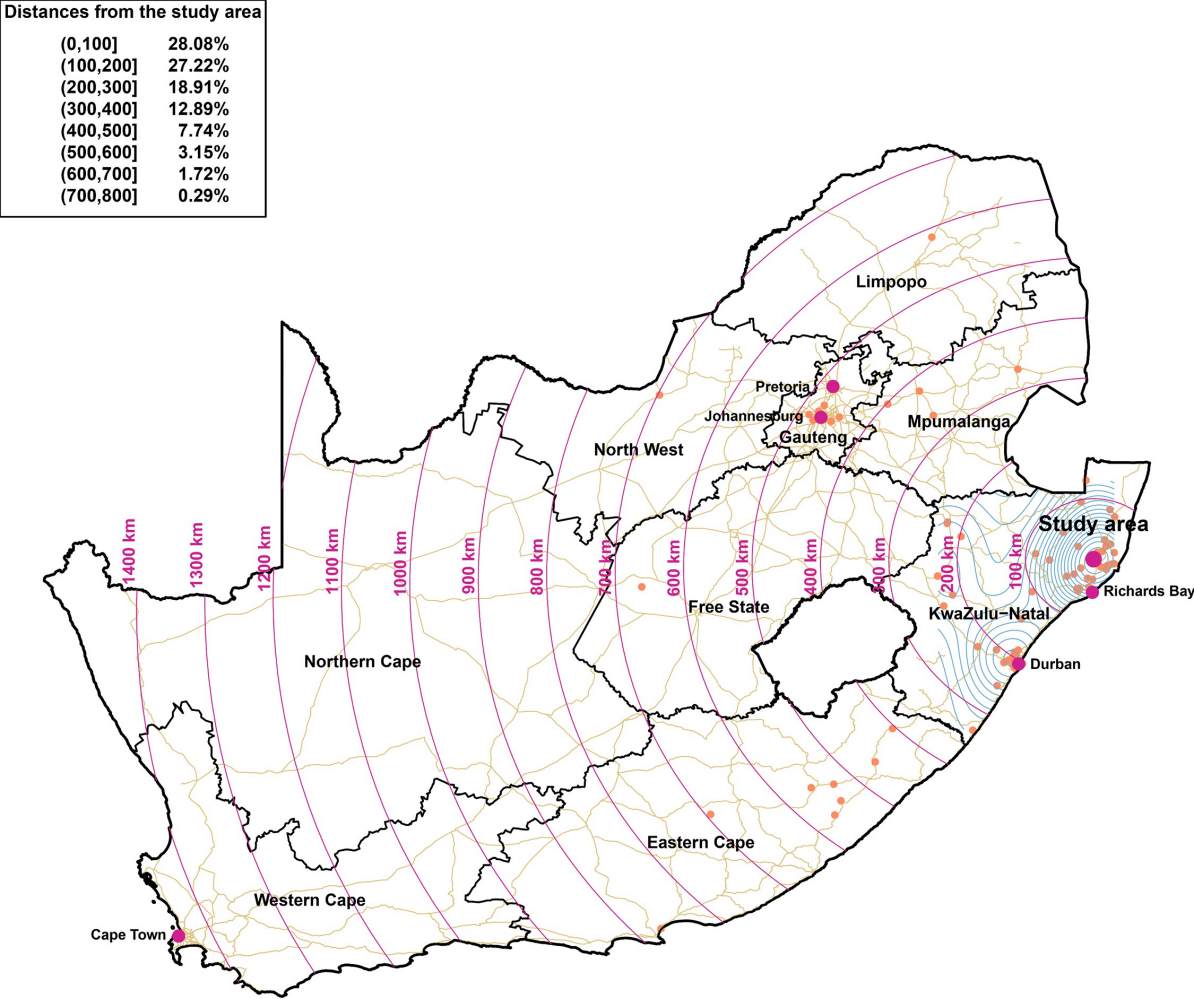
Map of the spatial distribution of the migration locations in South Africa (orange dots). The location of the Africa Health Research Institute rural study community is represented by a larger violet dot, whereas cities that are key migration destinations are represented by smaller violet dots. We mapped the motorways, trunk, and primary roads in South Africa, together with the boundaries of the nine South African provinces.

Of 594 women who out-migrated from the surveillance area during pregnancy or the first year postpartum and delivered outside the surveillance area, the destinations of 386 women could be determined based on the household members’ information, being shown in Fig 2. The majority were young women with a median age of 21 (IQR: 19–25) years. The most frequent destinations were Durban, Johannesburg, and the Richard’s Bay area, with slightly over 25% of the destinations being within 100 km of the study area.

### Reasons for external in- and out-migration

Of 3,944 pregnancies of women who had at least one migration episode, the reasons for external migration were provided and successfully linked for 2,885 (73.1%). When stratified by in- versus out-migration, the most frequent reason for migration into the surveillance area was accommodation (50.1%, 902/1,802), followed by employment (14.4%, 260/1,802) and education (13.2%, 238/1,802). Among those who out-migrated from the surveillance area, the most common reasons were also accommodation (35.7%, 387/1,083), employment (33.9%, 367/1,083), followed by education (18.8%, 204/1,083). Overall, pregnancy or delivery as the primary reason for migration was relatively low (5.4%) but significantly higher among those who in-migrated into the surveillance area (7.4%, 133/1,802) than those who out-migrated (2.2%, 24/1,083) (*p* < 0.001).

## Discussion

This study presented the mobility patterns of women during pregnancy and the first year postpartum and examined its effect on maternal mortality using one of the world’s largest longitudinal population-based cohorts based in rural South Africa. Approximately 20% of the pregnant women experienced external migration around the time of delivery; of these women, nearly 40% delivered outside the surveillance area. Whereas external migration did not affect HIV-negative postpartum women’s risk of mortality, HIV-positive women who externally migrated and delivered outside the surveillance area had a more than two times greater hazard of first-year mortality, suggesting that the adverse effect of migration is possibly linked to social and clinical factors pertaining to HIV-positive women.

The maternal mortality rate in the first year postpartum was high at 5.6 per 1,000 PY (and 831 per 100,000 live births), almost six times higher than those observed within 42 days of delivery. The observed mortality rate within 42 days of delivery was 130 per 100,000 live births, similar to the finding from the recent systematic review in South Africa [26]; however, little is known about the severity and causes of maternal deaths beyond the first 42 days of the postpartum period. In this study, more than 60% of the maternal deaths that occurred after 42 days were due to HIV- or TB-related conditions.

Other studies in South Africa have reported that postpartum women often travel back to their homes and family to deliver and receive care after delivery [13,20] or even leave their children with family members [21]. Women may relocate to look for employment [13,19,21] or because of marital dissolution [19] but often maintain their connections to family members. In our setting, participants were either physically residing in the households within the surveillance area or had maintained their relationships to the existing households as nonresidents throughout the pregnancy and postpartum period, with the majority (>80%) of women originating from the study area. However, high mobility into or out of the surveillance area was observed among both HIV-positive and HIV-negative peripartum women, especially young women in their early 20s. This finding is similar to the recent finding from Johannesburg which showed that the majority of peripartum mothers whose urban-rural migration was motivated by employment would travel back home in a rural area [21].

To our knowledge, this is the first study to demonstrate that HIV-positive peripartum women who experienced external migration away from the home environment are at heightened risk of mortality after delivery. One possible explanation for this finding is that HIV-positive women who externally migrated away from the study area, mainly seeking economic and educational opportunities in larger cities, might have had difficulty accessing care in a new environment. A recent mixed-method study in Swaziland reported that PLWH who were lost to follow-up from HIV care described mobility as the first factor that affected their falling out of the care [38]. It is also possible that HIV-positive women might have self-selected into migration, as seen among HIV-positive individuals in Malawi [19].

Mothers living with HIV often take ART to prevent HIV transmission to their infants at birth but may stop seeking care after safely delivering their babies [6,39]. Postpartum mothers, who already face the challenges of looking after a new baby, face a double burden by migrating [40] and being more likely to disengage from care and be lost to follow-up [4–8], putting them at heightened risk of adverse health outcomes. A recent systematic review showed that patients lost to follow-up in ART programs have a combined mortality of 46% [41]. In this study, HIV-positive mothers knew their status on average for 2 years at the time of pregnancy, and the majority of pregnant women who externally migrated delivered at hospitals or clinics, indicating that they were at least in contact with the health system at delivery. In Cape Town, South Africa, a study that linked routine electronic healthcare data and measured the movements and healthcare access of pregnant and postpartum women found that a substantial portion of HIV-positive postpartum women did not link to postpartum care and/or remained in care, especially if women were mobile around the country [42]. Special consideration of mobile peripartum women would be strongly needed regarding retention in care.

It is noteworthy that women living with HIV who returned to the study area did not experience an increased risk of mortality despite traveling and changing in residences. About half of those who traveled back to the study area did so to live with existing households and family members, seeking accommodation and family support, and they were likely familiar with the study area and social environment. Moreover, the HIV-negative women who externally migrated had a relatively lower hazard of mortality than those who had never migrated during the pregnancy and the first year postpartum. These findings could be explained by healthy migrant effects, with more than half of HIV-negative peripartum women migrating for employment or educational opportunities. On the contrary, the adverse effect of migration seemed to be more profound in peripartum women living with HIV. The findings emphasize the importance of targeted interventions for migrating HIV-positive women who might be far from family and the home environment, potentially through better access to care and retention in lifelong ART care after delivery. Further information on how migrating postpartum women access to and remain in care would be instructive.

The strength of this study is that we comprehensively geolocated changes in residency and were able to examine maternal mobility patterns using one of the largest population-based cohorts, including over 30,000 pregnancies in South Africa. We also measured the mortality rates beyond the first 42 days of delivery, providing the insights on the continued burden in the postpartum period. To reflect the scale-up of the ART program among pregnant women, we adjusted for the time period before and after 2010 but still observed a higher rate of mortality among HIV-positive pregnant women, especially those who externally migrated and delivered outside the surveillance area. This may persist despite the scale-up of universal ART and declining trends in maternal mortality [43]. Future studies need to assess the degree of engagement with the healthcare system and retention in care among migrating women living with HIV.

Notwithstanding these strengths, the study does have a number of limitations. First, a substantial proportion of participants had unknown HIV status at the time of delivery. To address the missing data on HIV status, we tried to fit multiple imputations using the clinical and sociodemographic factors included in this study; however, given the complex factors affecting the risk and timing of HIV acquisition, the model prediction and fit were quite poor. Nevertheless, the effect on model estimates would have been nondifferential, since known HIV-negative and HIV-positive women showed similar mobility patterns. Moreover, the mobility patterns among women with unknown HIV status were similar between those who had HIV-related deaths and the rest of these women. We ran the sensitivity analysis, in which HIV status was inferred from the causes of deaths predicted by the InterVA-4 model, which previously showed the high specificity and sensitivity for HIV-related deaths. Not only did HIV-related deaths present the strongest evidence for positive HIV status, but the overall findings remained robust, and even stronger in the sensitivity analysis. Second, we could not include detailed information on the frequency of visits or retention in ART care continuum among the participants in the postpartum period. We used a delivery setting as a proxy as to whether mothers had access to care during delivery. Although those who externally migrated had a slightly higher percentage of delivery at home, over 80% of mothers delivered at hospitals or clinics. Third, there could be unmeasured confounding or risk factors affecting maternal health outcomes for which we did not account in our analysis. Notably, we had limited data on the social or clinical context at the destinations. Our results highlight the importance of better understanding contextual factors and dynamics of HIV care among highly mobile young women living with HIV. Lastly, although the sample size of maternal deaths was relatively small because of the large number of women with unknown HIV status, we ran several sensitivity analyses under different assumptions for HIV status, for which our findings remained robust.

In conclusion, this study found that a substantial portion of peripartum women migrates around the time of delivery, and mobile HIV-positive peripartum women away from the home environment were at heightened risk of maternal mortality. These findings call for more efforts to develop and implement interventions for migrating women living with HIV to retain them in care, irrespective of their locations, and improve maternal health outcomes.

## Supporting information

S1 STROBE Checklist STROBE, Strengthening the Reporting of Observational Studies in Epidemiology. (DOC)

S1 Text Prespecified analysis plan. (DOCX)

S1 Table Sensitivity analysis for the association between mobility patterns and maternal mortality in the first year postpartum period among all eligible women. (DOCX)

S2 Table Mobility patterns by maternal HIV status at delivery. (DOCX)

S3 Table Baseline characteristics by maternal HIV status at delivery. (DOCX)

## Abbreviations

AHR: adjusted HR
AHRI: Africa Health Research Institute
ANC: antenatal care
ART: antiretroviral therapy
HR: hazard ratio
ICD-10: International Classification of Diseases, 10th revision
IQR: interquartile range
PLWH: people living with HIV
PY: person-years
STROBE: Strengthening the Reporting of Observational Studies in Epidemiology
TB: tuberculosis
