# Supplementary material for: Migration and first-year maternal mortality among HIV-positive postpartum women: A population-based longitudinal study in rural South Africa
Source: PLoS Med. 2020 Mar 31;17(3):e1003085. doi: 10.1371/journal.pmed.1003085 (PMC7108693; doi:10.1371/journal.pmed.1003085)
Supplement: S1 STROBE Checklist — (DOC) [file pmed.1003085.s001.doc]

STROBE Statement—Checklist of items that should be included in reports of ***cohort studies***

|  | Item No | Recommendation | Addressed on page number |
| --- | --- | --- | --- |
| **Title and abstract** | 1 | (*a*) Indicate the study’s design with a commonly used term in the title or the abstract | See title and abstract |
|  |  | (*b*) Provide in the abstract an informative and balanced summary of what was done and what was found | See abstract |
| Introduction | | |  |
| Background/rationale | 2 | Explain the scientific background and rationale for the investigation being reported | See introduction paragraphs 1-4 |
| Objectives | 3 | State specific objectives, including any prespecified hypotheses | See introduction paragraph 4 |
| Methods | | |  |
| Study design | 4 | Present key elements of study design early in the paper | See methods paragraphs 1-3 |
| Setting | 5 | Describe the setting, locations, and relevant dates, including periods of recruitment, exposure, follow-up, and data collection | See methods paragraphs 1-4 |
| Participants | 6 | (*a*) Give the eligibility criteria, and the sources and methods of selection of participants. Describe methods of follow-up | See methods paragraph 3 |
|  |  | (*b*) For matched studies, give matching criteria and number of exposed and unexposed | N/A |
| Variables | 7 | Clearly define all outcomes, exposures, predictors, potential confounders, and effect modifiers. Give diagnostic criteria, if applicable | See methods paragraphs 4-6 |
| Data sources/ measurement | 8* | For each variable of interest, give sources of data and details of methods of assessment (measurement). Describe comparability of assessment methods if there is more than one group | See methods paragraphs 1,2 |
| Bias | 9 | Describe any efforts to address potential sources of bias | See methods paragraph 7 |
| Study size | 10 | Explain how the study size was arrived at | See methods paragraphs 1-3 |
| Quantitative variables | 11 | Explain how quantitative variables were handled in the analyses. If applicable, describe which groupings were chosen and why | See methods paragraphs 4-6 |
| Statistical methods | 12 | (*a*) Describe all statistical methods, including those used to control for confounding | See methods paragraphs 7,8 |
|  |  | (*b*) Describe any methods used to examine subgroups and interactions | See methods paragraphs 7,8 |
|  |  | (*c*) Explain how missing data were addressed | See methods paragraph 8 |
|  |  | (*d*) If applicable, explain how loss to follow-up was addressed | N/A |
|  |  | (*e*) Describe any sensitivity analyses | See methods paragraph 7 |
| Results | | |  |
| Participants | 13* | (a) Report numbers of individuals at each stage of study—eg numbers potentially eligible, examined for eligibility, confirmed eligible, included in the study, completing follow-up, and analysed | See results paragraph 1 |
|  |  | (b) Give reasons for non-participation at each stage | N/A |
|  |  | (c) Consider use of a flow diagram | N/A |
| Descriptive data | 14* | (a) Give characteristics of study participants (eg demographic, clinical, social) and information on exposures and potential confounders | See results paragraph 1, Tale 1 |
|  |  | (b) Indicate number of participants with missing data for each variable of interest | See results Table 1 |
|  |  | (c) Summarise follow-up time (eg, average and total amount) | See results paragraph 2 |
| Outcome data | 15* | Report numbers of outcome events or summary measures over time | See results paragraph 2 |
| Main results | 16 | (*a*) Give unadjusted estimates and, if applicable, confounder-adjusted estimates and their precision (eg, 95% confidence interval). Make clear which confounders were adjusted for and why they were included | See results paragraphs 4,5 |
|  |  | (*b*) Report category boundaries when continuous variables were categorized | See methods paragraph 6 |
|  |  | (*c*) If relevant, consider translating estimates of relative risk into absolute risk for a meaningful time period | N/A |
| Other analyses | 17 | Report other analyses done—eg analyses of subgroups and interactions, and sensitivity analyses | See results paragraphs 6,7 |
| Discussion | | |  |
| Key results | 18 | Summarise key results with reference to study objectives | See discussion paragraphs 1-2 |
| Limitations | 19 | Discuss limitations of the study, taking into account sources of potential bias or imprecision. Discuss both direction and magnitude of any potential bias | See discussion paragraph 8 |
| Interpretation | 20 | Give a cautious overall interpretation of results considering objectives, limitations, multiplicity of analyses, results from similar studies, and other relevant evidence | See discussion paragraphs 3-7,9 |
| Generalisability | 21 | Discuss the generalisability (external validity) of the study results | See discussion paragraph 7 |
| Other information | | |  |
| Funding | 22 | Give the source of funding and the role of the funders for the present study and, if applicable, for the original study on which the present article is based | See funding |

*Give information separately for exposed and unexposed groups.

**Note:** An Explanation and Elaboration article discusses each checklist item and gives methodological background and published examples of transparent reporting. The STROBE checklist is best used in conjunction with this article (freely available on the Web sites of PLoS Medicine at http://www.plosmedicine.org/, Annals of Internal Medicine at http://www.annals.org/, and Epidemiology at http://www.epidem.com/). Information on the STROBE Initiative is available at http://www.strobe-statement.org.
