# Supplementary material for: Migration and first-year maternal mortality among HIV-positive postpartum women: A population-based longitudinal study in rural South Africa
Source: PLoS Med. 2020 Mar 31;17(3):e1003085. doi: 10.1371/journal.pmed.1003085 (PMC7108693; doi:10.1371/journal.pmed.1003085)
Supplement: S1 Text — (DOCX) [file pmed.1003085.s002.docx]

**Pre-specified analysis plan**

**Title of Proposal: Mobility patterns of peripartum women and their health outcomes by maternal HIV status**

**AHRI Faculty Member:** Frank Tanser

**Principal Investigator:** Hae-Young Kim

**Number of Co-investigators: 2**

**Category of letter submitted:** Other

**Funder if applicable:** None

**Intended project start date**: September 15^th^, 2017

**Expected end date:** December 31^st^, 2018

**Research Data Management: Will the study make sure of the following:** PIPSA

**Scientific hypothesis and specific research question/s (200-500 words) (Brief description of the proposal for non-specialists)**

HIV-positive women are often first diagnosed with HIV during pregnancy, followed by life-long initiation of antiretroviral therapy (ART). Several studies in South Africa have shown that pregnant women tend to relocate to stay with their extended family in a rural area and return for a few months after delivery. Such relocation can lead to discontinuation of care and worse health outcomes among HIV-positive postpartum women. However, limited studies have quantified the mobility patterns among HIV-positive peripartum women and potential association with maternal health outcomes. We hypothesize that a substantial proportion of both HIV-positive and HIV-negative peripartum women migrate during pregnancy and in the first-year postpartum period and that HIV-positive postpartum women who are mobile and externally out-migrate might be at the higher risk of adverse health outcomes due to challenges to access and link to care.

**Specific research aims and objectives (200-500 words)**

Our specific aims are: (1) to examine the migration patterns of peripartum women; and (2) to determine the association between migration patterns and the first-year maternal mortality by maternal HIV status.

**Research method/s (e.g. trial design; target population/s; sample size, use of clinical samples, bio-sampling)**

**Aim 1. Examine the migration patterns of peripartum women.** We will examine any change in the residential status among pregnant and postpartum women up to 1 year after delivery from 2000 to 2016. We will quantify and aggregate migration patterns in the overall study population as well as by maternal HIV status. External migration will be defined as moving into or out of the surveillance area. Maternal HIV status at the time of delivery will be determined using the annual AHRI HIV surveillance data. We will perform additional sensitivity analyses to determine HIV status for the missing data.

**Aim 2**. **Determine the association between migration patterns and first-year maternal mortality.** We will examine the association between migration patterns and first-year maternal mortality using a mixed-effects cox regression model. We will use the mortality data as ascertained via verbal autopsy with the closest caregiver of the deceased on an average of 6 months after the person’s death. The causes of deaths will be determined by the InterVA-4 model based on the set of symptoms, signs, and circumstances reported during the verbal autopsy interviews. All mothers will be censored 365 days after delivery if they are alive, or on the date of death if they decreased during the follow-up period. Other key confounders, including age, parity, education, and calendar year will be included in the adjusted model.

**Please indicate project outputs and corresponding time-frame (200-500 words)**

We expect that the current proposal will result in one peer-reviewed manuscript. We expect the study will be conducted from September 2017 to December 2018. This study will be conducted with a view to obtaining funding to design and implement interventions to improve maternal and infant health outcomes in a larger population.

**Likelihood of major advance in the field (200-500 words)**

This study will enhance our current understanding of the role of migration on maternal health outcomes, especially among HIV-positive peripartum women in rural South Africa. As female migration is increasing for seeking employment and/or education opportunities, it would be critical to better understand how migration affects maternal health outcomes in the postpartum period in order to design better interventions and improve health outcomes.

**Potential to analyse research synergies through partnerships/collaborations (200-500 words)**

The study will be conducted in collaboration with Prof Adrian Dobra at University of Washington. We are also planning to expand the current works using other health and demographic surveillance system (HDSS) datasets in South Africa.

**Potential to leverage additional resources and investments (200-500 words)**

Based on the study findings, we will apply to obtain funding to design and implement interventions to improve maternal health outcomes in a larger population scale in sub-Saharan Africa and other generalized HIV epidemic settings.

**Likelihood of advancing new products (200-500 words)**

The project will examine the impact of mobility on maternal health outcomes. We posit that the study findings will help better understand the impact of mobility at population-level and design other targeted interventions in the future.

**Impact on global public health and/or policy change (200-500 words)**

The findings will provide important insights into the potential relationships between mobility and adverse maternal health outcomes among pregnant and postpartum women in the hyperendemic rural South African setting. The findings will have important implications for HIV care among HIV-positive pregnant and postpartum women.

**Potential for public engagement activities – please comment on this point (200-500 words)**

As part of this study, we would not have a specified public engagement activity. However, the project results will be communicated with the community representatives, key stakeholders, the Department of Health, as well as presented at AHRI’s internal meetings.
