# Supplementary material for: Migration and first-year maternal mortality among HIV-positive postpartum women: A population-based longitudinal study in rural South Africa
Source: PLoS Med. 2020 Mar 31;17(3):e1003085. doi: 10.1371/journal.pmed.1003085 (PMC7108693; doi:10.1371/journal.pmed.1003085)
Supplement: S1 Table — (DOCX) [file pmed.1003085.s003.docx]

**S1 Table. Sensitivity analysis for the association between mobility patterns and maternal mortality in the first year postpartum period among all eligible women in 2000-2016***

| **Characteristic** | **Method 2†** |
| --- | --- |
|  | Adjusted Hazard Ratio (95% CI)‡ |
| **HIV status** |  |
| Positive vs Negative | **20.09 (13.23, 30.51)**** |
| **Migration** |  |
| Reside within DSA | Ref |
| Reside outside DSA | **1.76 (1.06, 2.93)*** |
| Migration & delivery within DSA | 0.47 (0.20, 1.11) |
| Migration & delivery outside DSA | 0.25 (0.06, 1.03) |
| **HIV positive status x Migration**^¶^ |  |
| Positive x Reside outside DSA | 1.66 (0.85, 3.26) |
| Positive x Migration and delivery within DSA | 2.70 (0.98, 7.41) |
| Positive x Migration and delivery outside DSA | **5.95 (1.31, 27.08)*** |
| **Parity** |  |
| 0 | Ref |
| 1 | **0.66 (0.46, 0.98)*** |
| 2+ | **0.44 (0.29, 0.68)**** |
| **Socioeconomic status (Household Asset)** |  |
| Poor | Ref |
| Poorest | **0.28 (0.20, 0.40)**** |
| Medium | **0.15 (0.10, 0.22)**** |
| Rich or Richest | **0.10 (0.06, 0.15)**** |

*p-value <0.05; **p-value <0.01

**†**Women with unknown HIV status whose death were attributable to AIDS or TB were considered as HIV-positive and the rest of mothers with unknown HIV status as HIV-negative.

‡Adjusted for all other covariates shown in each column. The model is also adjusted for age and year of delivery (3-knot cubic spline with knots at the 25^th^, 50^th^, and 75^th^ percentile for each parameter).
