# Supplementary material for: Migration and first-year maternal mortality among HIV-positive postpartum women: A population-based longitudinal study in rural South Africa
Source: PLoS Med. 2020 Mar 31;17(3):e1003085. doi: 10.1371/journal.pmed.1003085 (PMC7108693; doi:10.1371/journal.pmed.1003085)
Supplement: S2 Table — (DOCX) [file pmed.1003085.s004.docx]

*Participants with unknown HIV status were further stratified by having died due to AIDS or TB or not during the first year postpartum.

Abbreviations: DSA, Demographic Surveillance Area

| **Table S2. Mobility patterns by maternal HIV status at delivery** | | | | | | |
| --- | --- | --- | --- | --- | --- | --- |
| Mobility types | HIV-negative | HIV-positive |  | Unknown HIV status* | | |
|  |  |  |  | *Total* | Death due to AIDS or TB | Other |
|  | *n=10,958* | *n=3,339* |  | *n=15,994* | n=108 | n=15,886 |
| ***No external migration*** |  |  |  |  |  |  |
| Resided within DSA | 8526 (77.8) | 2479 (74.2) |  | 8329 (52.1) | 59  (54.6) | 8270  (52.1) |
| Resided outside DSA | 533  (4.9) | 238  (7.3) |  | 3173 (19.8) | 23  (21.3) | 3150  (19.8) |
| ***External migration*** |  |  |  |  |  |  |
| Delivery within DSA | 1253 (11.4) | 397  (11.9) |  | 2688 (16.8) | 14  (13.0) | 2674  (16.8) |
| Delivery outside DSA | 646  (5.9) | 225  (6.7) |  | 1804 (11.3) | 12  (11.1) | 1792  (11.3) |
