# Supplementary material for: Migration and first-year maternal mortality among HIV-positive postpartum women: A population-based longitudinal study in rural South Africa
Source: PLoS Med. 2020 Mar 31;17(3):e1003085. doi: 10.1371/journal.pmed.1003085 (PMC7108693; doi:10.1371/journal.pmed.1003085)
Supplement: S3 Table — (DOCX) [file pmed.1003085.s005.docx]

| **Table S3. Baseline characteristics by maternal HIV status at delivery** | | | |
| --- | --- | --- | --- |
|  | **HIV-negative** | **HIV-positive** | **Unknown HIV status** |
|  | n= 10,958 | n=3,339 | n= 15,994 |
| **Age at delivery, Median (Q1, Q3)** (years) | 22 (19, 29) | 27 (23, 32) | 24 (21, 29) |
|  | *n (%)* | *n (%)* | *n (%)* |
| **Time Period (years)** |  |  |  |
| 2003-2010 | 8059 (73.5) | 1718 (51.5) | 11630 (72.7) |
| 2011-2016 | 2899 (26.5) | 1620 (48.5) | 4361 (27.3) |
| **Ever attending ANC visits during the pregnancy** |  |  |  |
| Yes | 3668 (33.5) | 944 (28.3) | 4935 (30.9) |
| No | 1473 (13.4) | 267 (8.0) | 2023 (12.6) |
| Unknown | 5817 (53.1) | 2128 (63.7) | 9036 (56.5) |
| **Parity** |  |  |  |
| 0 | 5071 (46.3) | 610 (18.3) | 6543 (40.9) |
| 1 | 2286 (20.9) | 987 (29.6) | 4478 (28.0) |
| 2+ | 3601 (32.9) | 1742 (52.2) | 4973 (31.1) |
| **Social economic status (Household Asset)** |  |  |  |
| Poor or Poorest | 586 (5.4) | 164 (4.9) | 1223 (7.7) |
| Medium | 3234 (29.5) | 967 (29.0) | 4021 (25.3) |
| Rich | 2343 (29.6) | 1092 (32.7) | 4465 (28.1) |
| Richest | 3887 (35.5) | 1115 (33.4) | 6174 (38.9) |
| **Education** |  |  |  |
| No formal education or primary (grade 1-7) | 2238 (21.4) | 571 (17.2) | 2334 (15.8) |
| Secondary (grade ≥8) | 8204 (78.6) | 2759 (82.8) | 12424 (84.2) |
| **Marital Status** |  |  |  |
| Married | 1336 (12.4) | 146 (4.4) | 1161 (7.4) |
| Divorced, separated, widowed | 87 (0.8) | 33 (1.0) | 132 (0.8) |
| Never married | 9391 (86.8) | 3157 (94.6) | 14337 (91.7) |

Abbreviations: ANC, antenatal care
